# Supplementary figures and images for: Methyl donor deficient diets cause distinct alterations in lipid metabolism but are poorly representative of human NAFLD
Source: Wellcome Open Res. 2017 Aug 22;2:67. [Version 1] doi: 10.12688/wellcomeopenres.12199.1 (PMC5887079; doi:10.12688/wellcomeopenres.12199.1)

Supplementary Figure 1

A

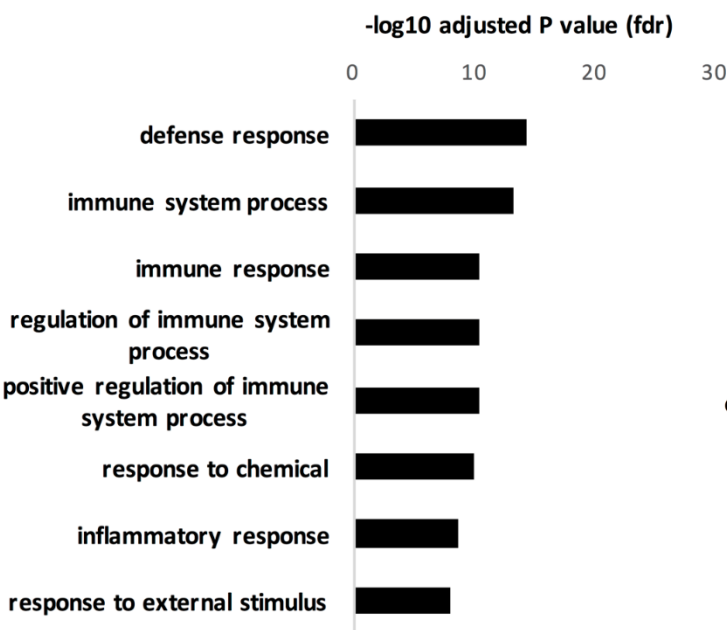

B

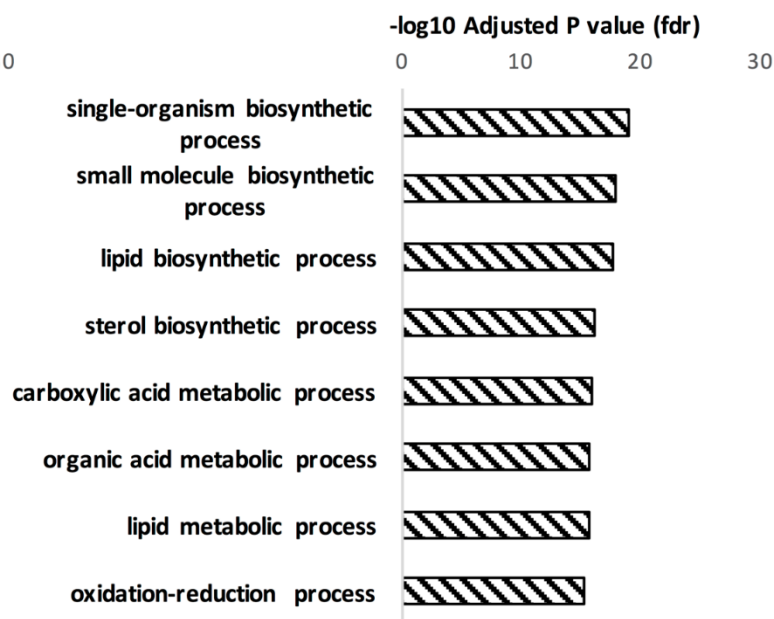

C

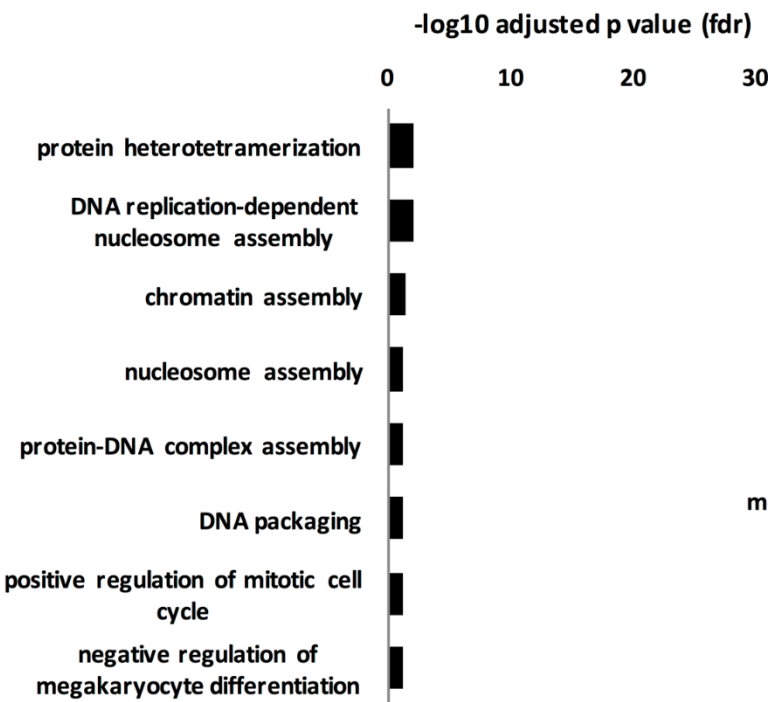

D

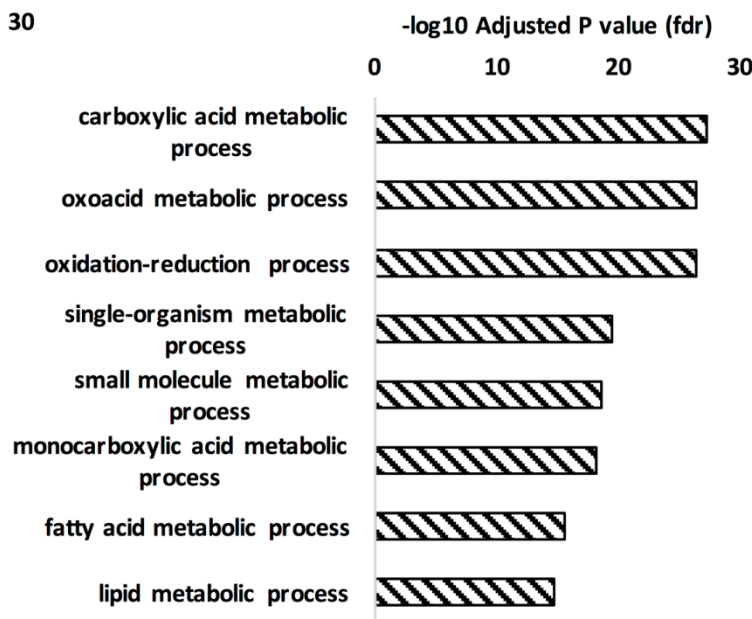

Supplement: Supplementary file 1 [file wellcomeopenres-2-13206-s0000.tgz › bd7b97c2-613d-455a-94ff-b56a9f4836db.pdf]
