# Supplementary material for: Methyl donor deficient diets cause distinct alterations in lipid metabolism but are poorly representative of human NAFLD
Source: Wellcome Open Res. 2017 Aug 22;2:67. [Version 1] doi: 10.12688/wellcomeopenres.12199.1 (PMC5887079; doi:10.12688/wellcomeopenres.12199.1)
Supplement: Supplementary file 2 [file wellcomeopenres-2-13206-s0001.tgz › 14173b0f-ca36-4fb8-961a-8f4eac5ea82e.pdf]

Supplementary Table 1: Dietary constituents. C: control diet; CDD: choline-deficient diet; MCDD: methionine- and choline-deficient diet. Manufacturers reference codes: CS, 518574; CDD, 518753; MCDD, 518810.

	Control (C)	Choline Deficient Diet (CDD)	Methionine Choline Deficient Diet (MCDD)
Kcal/gram	4.3	4.3	4.3
Kcal%			
Protein (as L-amino acids)	15	15	15
Carbohydrate	55	55	55
Fat	30	30	30
Choline Bitartrate (g/kg)	14.48	0	0
L-Methionine (g/kg)	1.7	1.7	0
