# Supplementary material for: Methyl donor deficient diets cause distinct alterations in lipid metabolism but are poorly representative of human NAFLD
Source: Wellcome Open Res. 2017 Aug 22;2:67. [Version 1] doi: 10.12688/wellcomeopenres.12199.1 (PMC5887079; doi:10.12688/wellcomeopenres.12199.1)
Supplement: Supplementary file 3 [file wellcomeopenres-2-13206-s0002.tgz › cf2c5328-4ac8-4cb7-b621-3511f8bcd2c5.pdf]

Supplementary Table 2: Primer sequences for qPCR validation.

Transcript	Forward	Reverse	UPL probe number
Gapdh	gggttcctataaatacggactgc	ccattttgtctacgggacga	52
Ldha	ggcactgacgcagacaag	tgatcacctcgtaggcactg	12
Scd1	ttccctcctgcaagctctac	cagagcgctggtcatgtagt	34
Aacs	cagtgctatgctgagattgagg	acacagccagggtcaagtg	1
Fasn	tccaccaaatccaacatgg	gttgtggaagtgcaggttagg	1
Lpl	ctcgctctcagatgccctac	aggcctggttgtgttgctt	95
Mlixpl	gacaccctgcacggactt	gtggttgctttgctcacctt	104
Acsl1	aaagatggctggttacacacg	cgataatcttcaaggtgccatt	46
Ces1d	cctcctacccgcctatgtg	ccttcctgttggtgaagagc	89
CES1f	agctggtagaaatcccgtcat	ttgtgggaatcaacaagcaa	5
Ces3b	ctggaggcatgattgtctca	atggtcgtgacaatcccact	3
Ces1g	ccccctcttggatctctga	cctctgggttttggtagcac	1
Pdk1	gcacattggaagcataaatcc	cctagcgttctcatagccatct	22
Ppia	Life Technologies (Paisley, UK) Taqman assay catalogue number # 4331182		
