# Supplementary material for: Methyl donor deficient diets cause distinct alterations in lipid metabolism but are poorly representative of human NAFLD
Source: Wellcome Open Res. 2017 Aug 22;2:67. [Version 1] doi: 10.12688/wellcomeopenres.12199.1 (PMC5887079; doi:10.12688/wellcomeopenres.12199.1)
Supplement: Supplementary file 4 [file wellcomeopenres-2-13206-s0003.tgz › d2dc07a5-e20a-48ed-97ef-764f9f8618de.pdf]

Supplementary Table 3: Top 100 dysregulated transcripts in CDD mice vs control mice

Gene Symbol	Gene Name	Entrez ID	lLog2 fold change vs Control	Adjusted P Value
Thrsp	thyroid hormone responsive	21835	-3.86	0.000265516
Lcn2	lipocalin 2	16819	3.28	0.030393419
Gpnmb	glycoprotein (transmembrane) nmb	93695	3.14	0.040528872
Gsta1	glutathione S-transferase, alpha 1 (Ya)	14857	2.93	0.003716367
Gsta1	glutathione S-transferase, alpha 1 (Ya)	14857	2.85	0.003903135
Ly6d	lymphocyte antigen 6 complex, locus D	17068	2.81	0.036028892
Acacb	acetyl-Coenzyme A carboxylase beta	100705	-2.68	0.000259422
Chrna4	cholinergic receptor, nicotinic, alpha polypeptide 4	11438	-2.67	0.000190259
Ly6d	lymphocyte antigen 6 complex, locus D	17068	2.66	0.043984713
Acss2	acyl-CoA synthetase short-chain family member 2	60525	-2.58	0.000303984
Pnpla3	patatin-like phospholipase domain containing 3	116939	-2.55	4.24E-05
Serpina4-ps1	serine (or cysteine) peptidase inhibitor, clade A, member 4, pseudogene 1	321018	-2.46	0.001972165
Chrna4	cholinergic receptor, nicotinic, alpha polypeptide 4	11438	-2.45	0.000190259
Cyp4a14	cytochrome P450, family 4, subfamily a, polypeptide 14	13119	2.44	0.00232634
Acly	ATP citrate lyase	104112	-2.36	0.000190259
Fdps	farnesyl diphosphate synthetase	110196	-2.30	0.000311749
Dbp	D site albumin promoter binding protein	13170	2.22	0.00278594
Slc16a5	solute carrier family 16 (monocarboxylic acid transporters), member 5	217316	2.19	0.001178228
Aacs	acetoacetyl-CoA synthetase	78894	-2.18	0.002443714
Cd63	CD63 antigen	12512	2.08	0.04452987

Mt1	metallothionein 1	17748	2.06	0.016173256
Elovl6	ELOVL family member 6, elongation of long chain fatty acids (yeast)	170439	-2.05	0.000578813
Elovl3	elongation of very long chain fatty acids (FEN1/Elo2, SUR4/Elo3, yeast)-like 3	12686	-2.02	0.001178228
Lgals3	lectin, galactose binding, soluble 3	16854	2.01	0.014404136
Aqp8	aquaporin 8	11833	-2.00	0.003114794
Fgf21	fibroblast growth factor 21	56636	1.99	0.045322923
Rdh11	retinol dehydrogenase 11	17252	-1.96	0.000197237
Trib3	tribbles homolog 3 (Drosophila)	228775	1.96	0.015852619
Mvd	mevalonate (diphospho) decarboxylase	192156	-1.95	0.001178228
Aqp8	aquaporin 8	11833	-1.92	0.000430423
Ccnd1	cyclin D1	12443	1.88	0.00255795
Mmp13	matrix metallopeptidase 13	17386	1.88	0.028799234
Ccnd1	cyclin D1	12443	1.82	0.001556373
Gsta1	glutathione S-transferase, alpha 1 (Ya)	14857	1.81	0.028799234
Dct	dopachrome tautomerase	13190	-1.79	0.000265516
Sqle	squalene epoxidase	20775	-1.77	0.004681862
S100a11	S100 calcium binding protein A11 (calgizzarin)	20195	1.76	0.015852619
Igfbp2	insulin-like growth factor binding protein 2	16008	1.76	0.004503458
Ear2	eosinophil-associated, ribonuclease A family, member 2	13587	1.71	0.016599162
Ear3	eosinophil-associated, ribonuclease A family, member 3	53876	1.69	0.014029385
Tmem86a	transmembrane protein 86A	67893	1.68	0.014174581
Ccnd1	cyclin D1	12443	1.67	0.013739961
Pcsk9	proprotein convertase subtilisin/kexin type 9	100102	-1.64	0.018832767
Ubd	ubiquitin D	24108	1.62	0.023100761

Uap1l1	UDP-N-acteylglucosamine pyrophosphorylase 1-like 1	227620	1.62	0.001178228
Rdh11	retinol dehydrogenase 11	17252	-1.61	0.000946057
Saa3	serum amyloid A 3	20210	1.58	0.010849298
Plin4	perilipin 4	57435	1.52	0.009511918
Ppp1r3b	protein phosphatase 1, regulatory (inhibitor) subunit 3B	244416	-1.51	0.041398543
Cd52	CD52 antigen	23833	1.49	0.020352777
Tm4sf4	transmembrane 4 superfamily member 4	229302	1.49	0.047585816
G6pc	glucose-6-phosphatase, catalytic	14377	-1.48	0.011058323
Lyz1	lysozyme 1	17110	1.47	0.024570972
Pmvk	phosphomevalonate kinase	68603	-1.47	0.002053525
Lss	lanosterol synthase	16987	-1.47	0.001206093
Ear2	eosinophil-associated, ribonuclease A family, member 2	13587	1.46	0.012495195
Dct	dopachrome tautomerase	13190	-1.45	0.000311749
Hes6	hairy and enhancer of split 6	55927	-1.44	0.000321576
Slc25a25	solute carrier family 25 (mitochondrial carrier, phosphate carrier), member 25	227731	-1.41	0.006394791
Mlxipl	MLX interacting protein-like	58805	-1.39	0.03064468
Acat2	acetyl-Coenzyme A acetyltransferase 2	110460	-1.39	0.003544893
Sc5d	sterol-C5-desaturase (fungal ERG3, delta-5-desaturase) homolog (S. cerevisae)	235293	-1.38	0.000190259
Slc30a10	solute carrier family 30, member 10	226781	-1.38	0.014404136
Mlxipl	MLX interacting protein-like	58805	-1.36	0.041556231
Tmie	transmembrane inner ear	20776	-1.35	0.02368705
Mmp12	matrix metallopeptidase 12	17381	1.35	0.026148843
Dhcr7	7-dehydrocholesterol reductase	13360	-1.34	0.002053525
Cdc20	cell division cycle 20	107995	1.33	0.041398543

Msmo1	methylsterol monoxygenase 1	66234	-1.30	0.019272363
Ces3b	carboxylesterase 3B	13909	-1.30	0.0317007
Mfsd7c	major facilitator superfamily domain containing 7C	217721	1.29	0.000197237
Stard4	StAR-related lipid transfer (START) domain containing 4	170459	-1.29	0.041398543
Ear4	eosinophil-associated, ribonuclease A family, member 4	53877	1.28	0.047585816
Adipor2	adiponectin receptor 2	68465	-1.27	0.015290696
Cbr3	carbonyl reductase 3	109857	1.27	0.04069306
Igfbp2	insulin-like growth factor binding protein 2	16008	1.26	0.015290696
Clec7a	C-type lectin domain family 7, member a	56644	1.26	0.026148843
Hes6	hairy and enhancer of split 6	55927	-1.26	0.013327131
Pnpla5	patatin-like phospholipase domain containing 5	75772	-1.25	0.00488006
Cd63	CD63 antigen	12512	1.24	0.018049651
Bcl2a1b	B cell leukemia/lymphoma 2 related protein A1b	12045	1.23	0.022093214
Cd68	CD68 antigen	12514	1.21	0.028799234
Vcam1	vascular cell adhesion molecule 1	22329	1.20	0.046089647
Fcer1g	Fc receptor, IgE, high affinity I, gamma polypeptide	14127	1.20	0.037183086
Bcl2a1d	B cell leukemia/lymphoma 2 related protein A1d	12047	1.20	0.017785151
Tlcd2	TLC domain containing 2	380712	-1.19	0.002155417
Hes6	hairy and enhancer of split 6	55927	-1.19	0.014276045
Paox	polyamine oxidase (exo-N4-amino)	212503	-1.19	0.000265516
Ttc39c	tetratricopeptide repeat domain 39C	72747	-1.17	0.040119653
Slamf9	SLAM family member 9	98365	1.17	0.0194431
Cyp4a12b	cytochrome P450, family 4, subfamily a, polypeptide 12B	13118	-1.15	0.017099634

Ces1b	carboxylesterase 1B	382044	1.15	0.043125539
Gm9883	predicted gene 9883	791409	-1.14	0.009511918
Sucnr1	succinate receptor 1	84112	-1.14	0.026148843
Abcd3	ATP-binding cassette, sub-family D (ALD), member 3	19299	-1.14	0.048650945
Sirpa	signal-regulatory protein alpha	19261	1.13	0.029143766
Haao	3-hydroxyanthranilate 3,4-dioxygenase	107766	-1.13	0.048650945
Tm7sf2	transmembrane 7 superfamily member 2	73166	-1.13	0.010000182
Scd1	stearoyl-Coenzyme A desaturase 1	20249	-1.12	0.009511918
Mgst3	microsomal glutathione S-transferase 3	66447	1.10	0.035439179
