# Supplementary material for: Methyl donor deficient diets cause distinct alterations in lipid metabolism but are poorly representative of human NAFLD
Source: Wellcome Open Res. 2017 Aug 22;2:67. [Version 1] doi: 10.12688/wellcomeopenres.12199.1 (PMC5887079; doi:10.12688/wellcomeopenres.12199.1)
Supplement: Supplementary file 5 [file wellcomeopenres-2-13206-s0004.tgz › ab818eb3-6c75-4509-a56c-6125e5331ee4.pdf]

Supplementary Table 4: Top 100 dysregulated transcripts in MCDD mice vs control mice

Gene Symbol	Gene Name	Entrez ID	Log2 fold change vs Control	Adjusted P value
Serpina1e	serine (or cysteine) peptidase inhibitor, clade A, member 1E	20704	-5.83	7.60E-08
Cyp4a12a	cytochrome P450, family 4, subfamily a, polypeptide 12a	277753	-4.77	1.24E-07
Hsd3b5	hydroxy-delta-5-steroid dehydrogenase, 3 beta- and steroid delta-isomerase 5	15496	-4.63	0.000319676
Ces3b	carboxylesterase 3B	13909	-4.50	7.69E-06
Cyp2b9	cytochrome P450, family 2, subfamily b, polypeptide 9	13094	4.05	1.31E-05
Ly6d	lymphocyte antigen 6 complex, locus D	17068	4.05	0.001137521
Cyp4a12b	cytochrome P450, family 4, subfamily a, polypeptide 12B	13118	-3.88	4.42E-06
Ly6d	lymphocyte antigen 6 complex, locus D	17068	3.84	0.001432616
Lcn2	lipocalin 2	16819	3.74	0.003405228
Thrsp	thyroid hormone responsive	21835	-3.72	6.18E-05
Cyp2a5	cytochrome P450, family 2, subfamily a, polypeptide 5	13087	3.71	0.000345532
Cyp2a5	cytochrome P450, family 2, subfamily a, polypeptide 5	13087	3.61	0.000479069
Serpina4-ps1	serine (or cysteine) peptidase inhibitor, clade A, member 4, pseudogene 1	321018	-3.58	3.33E-05
Elovl3	elongation of very long chain fatty acids (FEN1/Elo2, SUR4/Elo3, yeast)-like 3	12686	-3.55	9.35E-06
Nupr1	nuclear protein transcription regulator 1	56312	3.49	0.000553335
Mup4	major urinary protein 4	17843	-3.47	0.000753452
Scd1	stearoyl-Coenzyme A desaturase 1	20249	-3.37	4.14E-06
Mup5	major urinary protein 5	17844	-3.28	0.017504182
Mup20	major urinary protein 20	381530	-3.25	0.004614189

Cd63	CD63 antigen	12512	3.25	0.000961058
Wfdc3	WAP four-disulfide core domain 3	71856	3.19	0.004408789
G6pc	glucose-6-phosphatase, catalytic	14377	-3.12	2.49E-05
Serpina12	serine (or cysteine) peptidase inhibitor, clade A (alpha-1 antiproteinase, antitrypsin), member 12	68054	-3.06	0.002070405
Mup21	major urinary protein 21	381531	-3.04	0.000125954
Slpi	secretory leukocyte peptidase inhibitor	20568	3.00	0.000433325
Keg1	kidney expressed gene 1	64697	-2.96	8.69E-05
Trib3	tribbles homolog 3 (Drosophila)	228775	2.94	0.000364218
Dct	dopachrome tautomerase	13190	-2.91	4.25E-06
Gpnmb	glycoprotein (transmembrane) nmb	93695	2.89	0.013636195
Gsta1	glutathione S-transferase, alpha 1 (Ya)	14857	2.89	0.000642843
2810007J24Rik	RIKEN cDNA 2810007J24 gene	76971	-2.86	0.000437023
Cyp2a4	cytochrome P450, family 2, subfamily a, polypeptide 4	13086	2.86	0.001205536
Ccnd1	cyclin D1	12443	2.84	3.75E-05
Acss2	acyl-CoA synthetase short-chain family member 2	60525	-2.80	3.33E-05
Slc16a5	solute carrier family 16 (monocarboxylic acid transporters), member 5	217316	2.79	4.72E-05
Ccnd1	cyclin D1	12443	2.77	2.21E-05
Gsta1	glutathione S-transferase, alpha 1 (Ya)	14857	2.77	0.000863745
Mup2	major urinary protein 2	17841	-2.74	0.038027566
Cyp7b1	cytochrome P450, family 7, subfamily b, polypeptide 1	13123	-2.74	0.000754381
Tlcd2	TLC domain containing 2	380712	-2.72	0.001139948
Cyp4a14	cytochrome P450, family 4, subfamily a, polypeptide 14	13119	2.70	0.000240898
Acacb	acetyl-Coenzyme A carboxylase beta	100705	-2.70	3.92E-05

S100a11	S100 calcium binding protein A11 (calgizzarin)	20195	2.69	0.000326671
Egfr	epidermal growth factor receptor	13649	-2.68	1.01E-05
Chrna4	cholinergic receptor, nicotinic, alpha polypeptide 4	11438	-2.68	1.50E-05
Ces3b	carboxylesterase 3B	13909	-2.68	0.004433949
Cyp7a1	cytochrome P450, family 7, subfamily a, polypeptide 1	13122	2.64	0.000686107
Aqp8	aquaporin 8	11833	-2.63	0.000106559
Mup6	major urinary protein 6	620807	-2.59	0.012098321
Egfr	epidermal growth factor receptor	13649	-2.55	0.000247011
Slc25a25	solute carrier family 25 (mitochondrial carrier, phosphate carrier), member 25	227731	-2.55	3.33E-05
Cyp3a11	cytochrome P450, family 3, subfamily a, polypeptide 11	13112	2.54	0.00417431
Akr1b7	aldo-keto reductase family 1, member B7	11997	2.48	7.60E-08
Chrna4	cholinergic receptor, nicotinic, alpha polypeptide 4	11438	-2.48	2.36E-05
Pnpla3	patatin-like phospholipase domain containing 3	116939	-2.48	6.57E-06
Cdc20	cell division cycle 20	107995	2.47	0.000345091
Egfr	epidermal growth factor receptor	13649	-2.47	1.34E-05
Aqp8	aquaporin 8	11833	-2.44	2.21E-05
Anxa2	annexin A2	12306	2.37	0.001520745
Cyp2a5	cytochrome P450, family 2, subfamily a, polypeptide 5	13087	2.37	3.21E-05
Ces1e	carboxylesterase 1E	13897	-2.36	0.000534515
Mt1	metallothionein 1	17748	2.35	0.001638966
Mup2	major urinary protein 2	17841	-2.35	0.023564635
Tlcd2	TLC domain containing 2	380712	-2.31	1.01E-05
Haao	3-hydroxyanthranilate 3,4-dioxygenase	107766	-2.28	0.000197233

Dct	dopachrome tautomerase	13190	-2.28	5.97E-06
Ccnd1	cyclin D1	12443	2.27	0.000507114
Adam32	a disintegrin and metallopeptidase domain 32	353188	2.26	0.000404027
Cyp4f14	cytochrome P450, family 4, subfamily f, polypeptide 14	64385	-2.24	0.000274536
Fgf21	fibroblast growth factor 21	56636	2.23	0.005851945
App	amyloid beta (A4) precursor protein	11820	2.21	0.000200051
Igfbp2	insulin-like growth factor binding protein 2	16008	2.20	0.00022238
C6	complement component 6	12274	-2.18	8.51E-05
Rgs16	regulator of G-protein signaling 16	19734	-2.18	0.003408252
Haao	3-hydroxyanthranilate 3,4-dioxygenase	107766	-2.17	0.000376645
Tlcd2	TLC domain containing 2	380712	-2.17	1.41E-05
Inmt	indolethylamine N-methyltransferase	21743	-2.17	0.001358661
Haao	3-hydroxyanthranilate 3,4-dioxygenase	107766	-2.14	0.000270365
Ddit3	DNA-damage inducible transcript 3	13198	2.14	0.000848135
Mat2a	methionine adenosyltransferase II, alpha	232087	2.14	0.000121488
Gnmt	glycine N-methyltransferase	14711	-2.12	0.001276227
Htatip2	HIV-1 tat interactive protein 2, homolog (human)	53415	2.12	2.40E-05
Htatip2	HIV-1 tat interactive protein 2, homolog (human)	53415	2.11	2.21E-05
Fos	FBJ osteosarcoma oncogene	14281	-2.08	0.00404462
Macrod1	MACRO domain containing 1	107227	-2.08	2.32E-05
Ttc39c	tetratricopeptide repeat domain 39C	72747	-2.07	0.001090088
Lyz2	lysozyme 2	17105	2.07	0.018695846
C8b	complement component 8, beta polypeptide	110382	-2.06	0.000148242
Gm6484	predicted gene 6484	624219	2.06	0.004118688

Apoa4	apolipoprotein A-IV	11808	2.05	0.003331964
Srd5a1	steroid 5 alpha-reductase 1	78925	-2.03	0.000167264
Ces1f	carboxylesterase 1F	234564	-2.03	0.001917118
C6	complement component 6	12274	-2.03	2.20E-05
Afmid	arylformamidase	71562	-2.02	9.62E-05
Gck	glucokinase	103988	-2.02	0.004419283
Lgals3	lectin, galactose binding, soluble 3	16854	2.02	0.002722111
Cdc20	cell division cycle 20	107995	2.00	0.000393359
Psat1	phosphoserine aminotransferase 1	107272	1.99	3.33E-05
Susd4	sushi domain containing 4	96935	-1.99	0.000584512
C8a	complement component 8, alpha polypeptide	230558	-1.99	0.0052156
