# Supplementary material for: Methyl donor deficient diets cause distinct alterations in lipid metabolism but are poorly representative of human NAFLD
Source: Wellcome Open Res. 2017 Aug 22;2:67. [Version 1] doi: 10.12688/wellcomeopenres.12199.1 (PMC5887079; doi:10.12688/wellcomeopenres.12199.1)
Supplement: Supplementary file 6 [file wellcomeopenres-2-13206-s0005.tgz › d7a38c1d-2e64-4806-8558-159ea53c1473.pdf]

Supplementary Table 5: Characteristics of patients used for NAFLD comparison with CDD and MCDD transcriptome. Brackets indicate interquartile range. NR= not reported

	**Controls**		**Steatosis**		**NASH**	
	Ahrens et al	Lake et al	Ahrens et al	Lake et al	Ahrens et al	Lake et al
n	18	19	12	10	15	9
BMI	24 (21-26)	NR	50(47-55)	NR	49 (44–56)	NR
steatosis	0	<10%	30% (20-70)	>10% hepatocytes	75% (70-85)	>5% hepatocytes
NAS score	0 (0–0)	0	2(1-3)	<3	5 (5-6)	>3
Fibrosis score	0 (0–0)	0	0 (0–1)	NR	0 (0–1)	>1
