# Supplementary material for: Methyl donor deficient diets cause distinct alterations in lipid metabolism but are poorly representative of human NAFLD
Source: Wellcome Open Res. 2017 Aug 22;2:67. [Version 1] doi: 10.12688/wellcomeopenres.12199.1 (PMC5887079; doi:10.12688/wellcomeopenres.12199.1)
Supplement: Peer review history

# Peer review: Referee response for version 1

**DOI:** 10.21956/wellcomeopenres.13206.r27961

**Published:** 2017-11-28

**Authors:** David A. Sheridan, Paula Boeira

**Affiliations:** 1 Institute of Translational and Stratified Medicine (ITSMED), Plymouth University, Plymouth, UK; 2 Institute of Translational and Stratified Medicine (ITSMED), Plymouth University, Plymouth, UK

This is an interesting study suggesting a novel mechanism for hepatic lipid accumulation in methyl donor deficient diets. Non-alcoholic fatty liver disease (NAFLD) is increasingly diagnosed worldwide and is considered to be the most common liver disorder in Western countries. There is an urgent need for more studies defining the molecular pathways of NAFLD pathology and representative rodent models that mimic such a complex disease. For that reason the relevance of this study is clear. The article is well written, the methods well explained and the results clearly outlined.

 The authors are aware that this rodent model lacks key features of NAFLD in humans such as obesity, type 2 diabetes and insulin resistance. Both methyl donor deficient diets did not induce weight gain and resulted in hepatic steatosis without insulin resistance.  It is important to appreciate as the authors do in the introduction, that there are other diet induced obesity mouse models of NASH (e.g. c57BL/6) that can be induced with an obesogenic diet, although histological features of necroinflammation and fibrosis take longer to appear. The main advantage of nutrient deficient diets is that the liver injury is occurring more rapidly and predictably, which has led to widespread use of these dietary deficit models to study NASH. Whilst it is of importance to describe the transcriptome differences between MCDD/ CDD diets and human NAFLD, it would also have been of interest to compare MCDD CDD models to diet induced obesity mouse models as well.

 It is also important to recognise that the phenotype of MCDD CDD diets is different from human NAFLD, with significant weight loss and loss of liver volume, with a hyper catabolic state and lack of insulin resistance. Figure 1 illustrates clearly the significant weight loss and loss of liver volume in MCDD compared to CDD, whilst developing more severe liver injury and fibrosis.

 The lipid pathways analysis again reveals more marked changed in MCDD than CDD mouse models, but similarities in suppression of lipid synthesis genes, and increased expression of genes involved in inflammatory pathways.  

 Particular caution should be made with the comparison of transcriptional data from both diets were to published human genomics data. One concern is that these human gene sets are obtained from liver biopsies during bariatric surgery, thus typically these patients undergo bariatric surgery following a strict calorie controlled diet specially designed to reduce liver volume at the time of surgery. This could have a high influence on the gene analysis results. In addition, another factor that potentially influenced the cross species comparison was the small study population. Selection of larger, non-bariatric surgery NASH liver biopsies should be considered for comparison to the human transcriptome reference data. Further comparison of MCDD / CDD to DIO mouse models and larger human NASH liver transcriptomes would be valuable.

 The authors used a dataset of mixed hepatitis C and alcohol-induced hepatocellular carcinoma (HCC) samples to investigate the malignant processes present in NALFD. However this comparison is inaccurate, since several mechanisms that are involved in HCV and ALD are not present in NAFLD-induced HCC. There was no correlation between each dietary intervention to HCC.

 In conclusion, the paper suggests a novel mechanism where impaired VLDL assembly due to supressed triglyceride hydrolysis proteins is involved in methyl donor deficient liver injury. Furthermore, this study demonstrates the limitations of methyl donor deficient diet rodent models to investigate NAFLD. Further research is necessary to develop more accurate models that better mimic the disease spectrum, in order to provide both increased mechanistic understanding and identification of novel therapeutic approaches.

We have read this submission. We believe that we have an appropriate level of expertise to confirm that it is of an acceptable scientific standard.

# Peer review: Referee response for version 1

**DOI:** 10.21956/wellcomeopenres.13206.r25266

**Published:** 2017-09-04

**Authors:** John F. Dillon, Paul N Brennan

**Affiliations:** 1 Division of Molecular and Clinical Medicine, Ninewells Hospital and Medical School, University of Dundee, Dundee, UK

We read with interest the paper submitted by Lyall and colleagues. Non-Alcoholic Fatty Liver Disease (NAFLD) is a truly global health issue with significant associated morbidity and mortality, the only currently available therapeutic intervention is weight loss and exercise. The development of new therapeutics is hampered by the lack of animal models that have been validated against human disease for both NAFLD and the associated phenotype; NASH (Non-Alcoholic SteatoHepatitis) and attempts to validate models are most welcome.  

 The paper described the expected steatosis and fibrosis within both the CDD and MCDD cohorts, which superficially resembles NASH, but as in human disease histo-pathological similarity does not imply common aetiology. The authors rightfully comment on the inherent physiological and phenotypic confounders within the models compared with human NAFLD subjects. NAFLD is principally, albeit not exclusively, characterised by peripheral insulin resistance, obesity and hepatic accumulation of triglycerides. Within the MCDD rodents there was significant weight loss and insulin sensitivity, while the CDD mice whilst developing hepatic steatosis, did not demonstrate insulin resistance. 

 Within the gene analysis groups there were small numbers interrogated, likely related to the cost of analysis being prohibitive. Within this cohort however; there was marked upregulation of "immune system process" and "inflammatory process" with significant concordance between both groups.  

 Interestingly, dominant pathways of hepatic fatty acid fate were relatively unperturbed; although there is invariably some discordance between murine models and humans, particularly related to peroxisome metabolism.

 Whilst the study attempted to use closely modelled comparators; there were difficult variables with which the authors had to contend. This relates in particular to the HCC cohort; which comprised of composite HCC-related to HCV and ALD, which while similar have understated intrinsic differences. The CDD and MCDD transcriptomes grossly demonstrated very little relation to human NAFLD. Of the genes that bore commonality, the expression profiles were often oppositional and of no statistical significance.  The observation of changes in PNPLA3 expression, which is the gene most strongly associated with Human disease, should not be taken as evidence of similarity between the models and human disease, the activity of PNPLA3 is not specific to NAFLD but important in regulating lipid handling and appears in other liver diseases to have an impact on adaptive responses to oxidative stress

 It is difficult to extrapolate the findings in relation to s-adenosylmethionine (SAMe). Recent work has suggest amelioration of steatotic features within certain murine models but it is interesting that there was no metabolic variability in SAMe mediators. Certainly the work presents a novel mechanism for methyl donor deficient liver injury in a murine model affecting impaired VLDL particulate assembly as a consequence of suppressed triglyceride hydrolysis proteins. It does not however; bear significant transcriptional resemblance to human NAFLD or HCC.

 This study does definitively demonstrate that whilst there are common histological features between murine MCDD, CDD and human NAFLD; there are obviously inherent differences in how these pertain to transcriptomic expression profiles. This would of course suggest that there are conceivably alternative pathways which produce identical histological outcomes. Finally, the paper seeks to raise important concerns about the limitations of both CDD and MCDD models in replicating human NAFLD and efforts should continue to focus on developing more accurate models for future studies to utilise.

We have read this submission. We believe that we have an appropriate level of expertise to confirm that it is of an acceptable scientific standard.
